# Quality Tolerance Limits: Framework for Successful Implementation in Clinical Development

**DOI:** 10.1007/s43441-020-00209-0

**Published:** 2020-09-03

**Authors:** Ruma Bhagat, Lukasz Bojarski, Soazig Chevalier, Dagmar R. Görtz, Stéphanie Le Meignen, Marcin Makowski, Patrick Nadolny, Marion Pillwein, Melissa Suprin, Sabine Turri

**Affiliations:** 1 Roche, 1 DNA Way, South San Francisco, CA 94404 USA; 2 AstraZeneca, Postępu 14, 02-676 Warsaw, Poland; 3 Sanofi, 1 avenue Pierre Brossolette, 91385 Chilly Mazarin, France; 4 Janssen-Cilag GmbH, Johnson & Johnson Platz 1, 41470 Neuss, Germany; 5 Bristol Myers Squibb, 3 rue Joseph Monier, 92506 Rueil-Malmaison, France; 6 UCB Biosciences GmBH, Alfred-Nobel-Strasse 10, 40789 Monheim am Rhein, NRW Germany; 7 Allergan, 2525 Dupont Drive, Irvine, CA 92612 USA; 8 Merck KGaA/EMD Serono, Frankfurterstrasse 250, 64293 Darmstadt, Germany; 9 Pfizer, Inc., Eastern Point Road, Groton, CT 06340 USA; 10 Novartis Pharma S.A.S., 92506 Pueil-Malmaison Cedex, France

**Keywords:** Quality tolerance limits, QTL, Quality management system, QMS, Key risk indicators, KRI

## Abstract

The International Council for Harmonisation (ICH) E6(R2) (International Council for Harmonisation (ICH). ICH harmonised guideline: integrated addendum to ICH E6(R1): guideline for good clinical practice E6(R2). 2016. https://database.ich.org/sites/default/files/E6_R2_Addendum.pdf. Accessed 5 Dec 2019) introduced Quality Tolerance Limits (QTLs) to the industry, and in doing so, modernized quality control for clinical trials. QTLs provide measured feedback on clinical trial parameters previously only used by statistical and clinical functions to track trial progress toward endpoints. Elevating these measures as part of the Quality Management System (QMS) provides greater visibility across clinical trial functions and the enterprise as well as to measures that are important indicators of the state of participant protection and reliability of trial results. In support of this new requirement, TransCelerate developed a framework to guide industry sponsors and their agents in implementing QTLs. This QTL Framework is intended to aid industry’s ability to improve the quality of clinical research through the implementation of QTLs in a way that helps protect trial participants and reliability of trial results while meeting Health Authority (HA) expectations. The framework is intended to maximize efficiency and minimize confusion in the implementation of QTLs. The framework includes proposed approaches for implementation of QTLs for a clinical trial as defined in Section 5.0.4 and 5.0.7 of ICH E6(R2) (International Council for Harmonisation (ICH). ICH harmonised guideline: integrated addendum to ICH E6(R1): guideline for good clinical practice E6(R2). 2016. https://database.ich.org/sites/default/files/E6_R2_Addendum.pdf. Accessed 5 Dec 2019) and considerations for setting thresholds.

**Electronic supplementary material:**

The online version of this article (10.1007/s43441-020-00209-0) contains supplementary material, which is available to authorized users.

## Introduction

In the past decade, clinical development regulations have moved with the pharmaceutical industry to modernize clinical development and embrace the paradigms of risk-based quality management. A major revision to Good Clinical Practice (GCP) Guidelines occurred in November 2016 with the publication of the International Council for Harmonisation of Technical Requirements for Pharmaceuticals for Human Use (ICH) E6(R2) guidelines [1], later adopted by various Health Authorities (HA). Section 5.0 Quality Management was added to the guidelines with the introduction of a risk-based approach to quality management at the protocol and system levels. The guidelines included the requirement to establish Quality Tolerance Limits (QTLs) to guide clinical trial quality proactively by controlling for risks and allowing for corrective actions to be taken during the conduct of the trial to avoid later quality issues. The introduction of QTLs challenged sponsors with interpreting and operationalizing the guidance.

The QTL process described in this framework includes three stages: Define, Monitor, and Report. The Define stage, occurring after availability of a draft protocol and before enrollment of the first participant, includes defining the parameters and thresholds for QTLs. This stage also includes development of a QTL monitoring plan to define timeframe and frequency of reviews and data sources for monitoring. During the Monitor stage, while the clinical trial has participants in the clinic, the framework calls for periodic reporting according to the monitoring approach. Any QTL deviations from the predefined threshold(s) would be investigated and corrected as needed. After the trial ends, a summary report of QTL deviations and associated preventive and/or corrective actions would be generated. Under the framework, the highlights or important QTL deviations and associated actions would be included in the clinical study report (CSR). Implementing QTLs according to this framework is consistent with ICH E6 and industry best practice for measuring and monitoring clinical trial quality.

### ICH Guideline Reference

ICH E6(R2) indicates that QTLs be established to “identify systematic issues that can impact subject safety or reliability of trial results” and that important deviations from the predefined QTLs and associated remedial actions taken are reported in the CSR [1].

### Development of the TransCelerate QTL Framework

Following the release of ICH E6(R2), TransCelerate’s Risk-Based Monitoring initiative produced a position paper exploring this new concept and providing implementation considerations for establishing QTLs and risk reporting in the CSR [2].

In response to learnings from implementation of QTLs since the position paper was written, TransCelerate’s Interpretations of Guidances & Regulations (IGR) initiative identified a team of subject matter experts (SMEs) from 11 of its member companies to revisit the ICH E6(R2) guideline with respect to QTLs. This document is the result of discussions sharing best implementation practices for effective and efficient implementation of QTLs.

## How QTLs Fit in a Quality Management System

Quality by Design (QbD) principles underpin a risk-based Quality Management System (QMS). QTLs are part of that risk-based approach. They are an added control for risks to factors that are critical to quality (i.e., CtQ factors). In clinical trials, CtQ factors are those with the potential to impact participant protection and/or the reliability of trial results. These may include the following:

- Primary objective
- Safety objectives
- Patient eligibility
- Investigational product exposure

CtQ factors related to critical data and processes are also described in ICH E8(R1) General Considerations for Clinical Studies (draft version) [3]. Section 3.2 of the draft guidance states that these quality factors are considered to be critical because, if their integrity were to be undermined by errors of design or conduct, the reliability or ethics of decision-making would also be undermined.

Therefore, at the time of protocol design, CtQ consideration is foundational to ensuring that trials are designed with quality built in. In parallel, sponsors should define appropriate risk management strategies to protect trial participants and the reliability of trial results (i.e., Integrated Quality Risk Management Plans enabling risk-based monitoring strategy). This includes the use of controls like QTLs and Key Risk Indicators (KRIs).

**Fig. 1:**
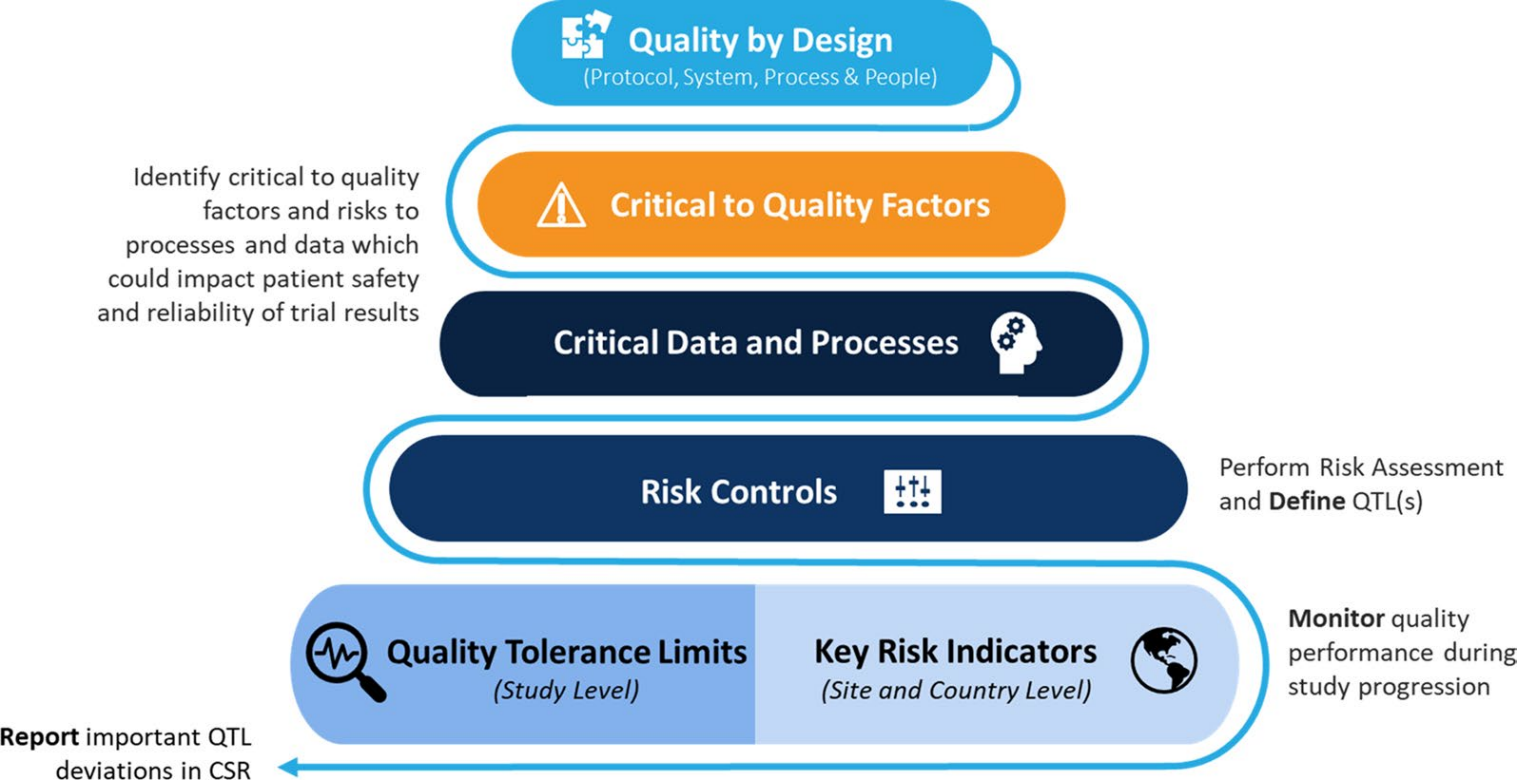
Risk-Based Quality Management Components. *CSR* Clinical Study Report; *QTL* Quality Tolerance Limit

As noted in Fig. 1, QbD is present throughout a trial, starting with the protocol design and the identification of CtQ factors, through to the monitoring of data during the conduct of the trial, and concluding with analysis of the impact of important deviations in the CSR.

## QTL Definition and Purpose

A QTL is a level, threshold, or value associated with a parameter which is critical to quality. QTLs are set for risks identified at the trial level. A deviation from a threshold during the conduct of the trial may indicate a systematic issue that could impact participants’ safety or reliability of trial results. QTLs should be defined with the protocol and no later than the first participant’s first visit (FPFV).

Performance should be monitored against the predefined QTLs and assessed on a regular basis throughout the course of a trial. Additionally, any trend indicating that a QTL deviation may occur could trigger an evaluation to assess if action is needed to avoid a potential deviation from the predefined QTL.

Based on the systematic nature of the issues discovered through the use of QTLs, some of the remediations would benefit future studies by identifying risks and ways to control them proactively. This can be addressed in the risk review part of the risk management process [4].

Early action thresholds (i.e., secondary limits) could be specified for QTL parameters to provide study teams with early opportunities to mitigate risks to participant safety or reliability of trial results and avoid a QTL deviation. This added limit allows study teams to intervene before an important deviation from a QTL is observed, if desired.

### Number of QTLs

In keeping with a risk-based approach, the number of QTLs should be commensurate with level of risk associated with the protocol. QTLs should be carefully selected and, ideally, aligned with CtQ factors. Too many QTLs will dilute the importance of each QTL and the amount of time available to spend on controlling factors that contribute to each one.

### Relationship Between QTLs and KRIs

QTLs and KRIs help control risks identified early in the clinical development process. Both are defined and measured to manage factors that are critical to quality during the conduct of the trial. In some cases, QTLs and KRIs may share the same parameter (e.g., proportion of participants with protocol deviations on eligibility criteria or proportion of participants with premature discontinuation). KRIs and QTLs differ in that KRIs are typically measured at the site level to inform site monitoring activities, while QTLs are a higher-level indication of overall quality in a trial. An example of a potential relationship between a CtQ factor, a QTL, and a KRI is shown in Table 1.

**Table 1:** Example CtQ Factor and Associated QTL and KRI.

CtQ factor	QTL	KRI
Withdrawal criteria and trial participant retention	Percentage or number of participants with withdrawal of informed consent	Presence of participants at site who withdrew consent

*CtQ* critical to quality, *KRI* key risk indicator, *QTL* quality tolerance limit

Additionally, a QTL deviation at the trial level, for example participants with withdrawal of informed consent at the trial level, is not necessarily coincident with a KRI deviation at the site or country level.

On the contrary, if the QTL deviation occurs, it is anticipated that an equivalent KRI deviation will occur for multiple sites or countries. Either scenario will require an evaluation of the issue and mitigation activities at the appropriate level.

Finally, in a risk-based approach, some KRIs may not be suitable as QTL parameters. The most important factors across the trial warrant QTLs. Other supporting quality indicators are better suited for KRIs. For example, metrics related to compliance (e.g., Site Trial Master File and Investigator Site Form completeness metrics) require oversight to ensure the integrity of the trial, but may not be as significant to human subject protection or reliability of trial results as QTLs.

## QTL Process

**Fig. 2:**
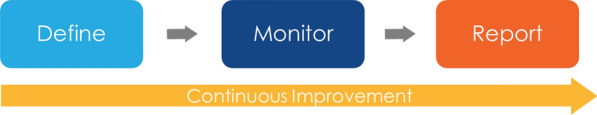
Quality Tolerance Limit (QTL) Framework—Process Overview.

**Fig. 3:**
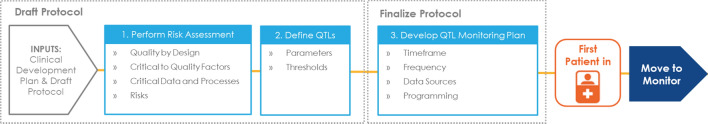
Define QTLs Process Map.

**Fig. 4:**
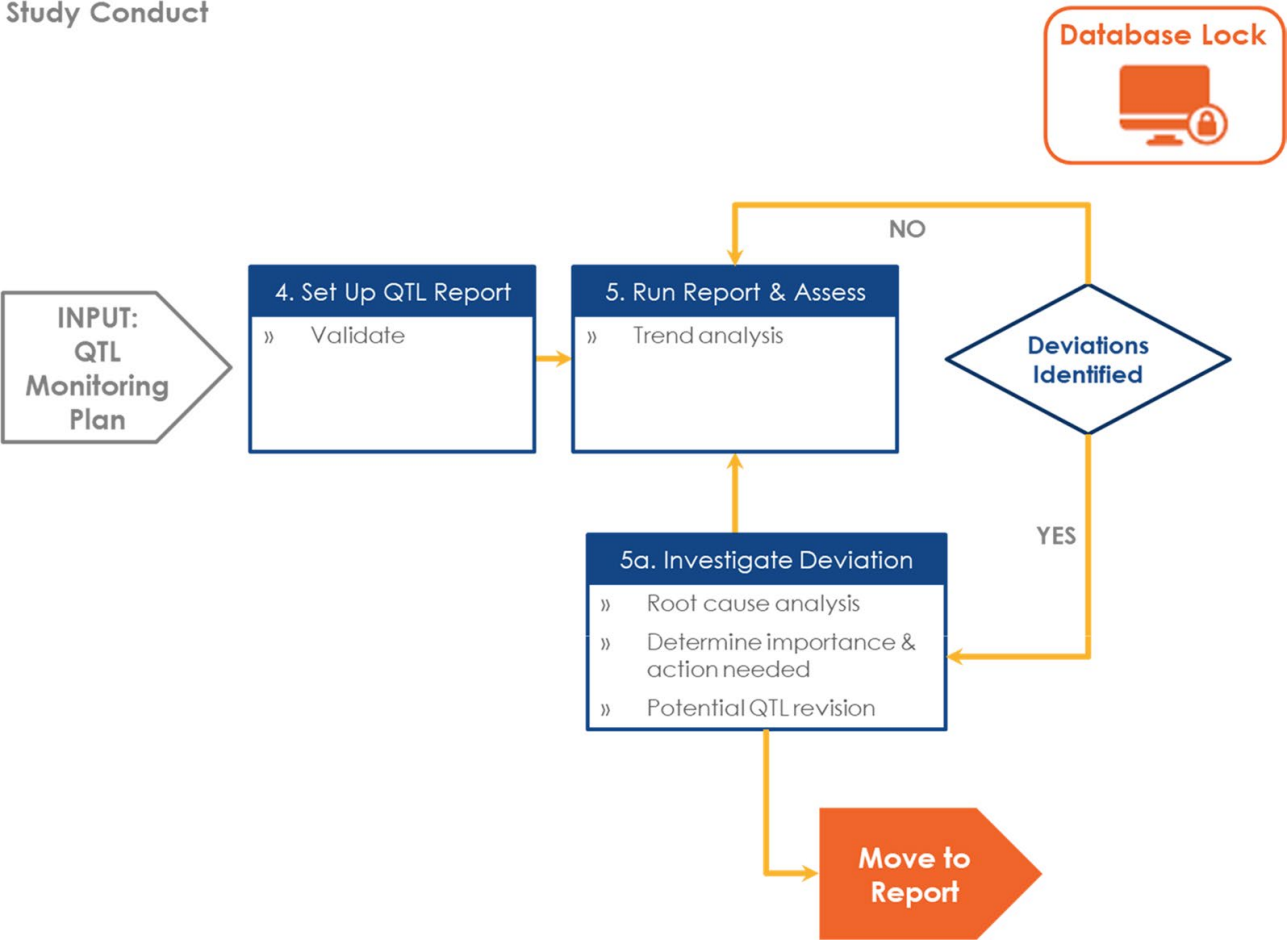
Monitor QTLs Process Map.

**Fig. 5:**
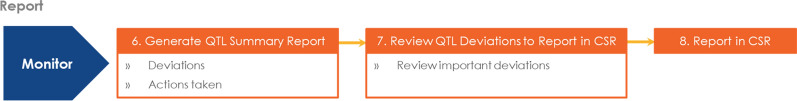
Report on QTL Process Map.

QTLs act as controls for risk and are part of the Clinical Trial Quality Risk Management process. This QTL Framework includes a process for defining, monitoring, and reporting QTLs, which corresponds to the set-up, conduct, and closeout phases of a clinical trial (Fig. 2). A separate detailed process map and considerations for each stage follow (Figs. 3, 4, and 5).

Defining, monitoring, and reporting of QTLs is typically a cross-functional process involving some or all of the following groups: Clinical Development, Clinical Operations, Biostatistics, Medical Monitoring, Medical Writing, Data Management, Clinical Supplies, Pharmacovigilance, Clinical Development Quality Assurance, and Centralized Monitoring. Different companies employ different models for resourcing QTL development. Whether using a facilitated model in which a quality professional or study management is leading the process or a functional model in which members of the study team are leading QTL activities, clinical and statistical functional experts should be engaged throughout the process.

If the program uses outsourcing, the sponsor may choose to involve the contract research organization (CRO), or other involved organizations, in management of QTLs. If management of QTLs is outsourced, the sponsor maintains accountability for the process. In addition, QTL-related activities must be documented in order to demonstrate compliance.

### Define Process Stage

The Define stage of the QTL process starts after a draft protocol is available. The following steps are included in the Define stage (Fig. 3):

1. Perform Risk Assessment—identify CtQ factors and associated risks to critical processes and data, which could impact patient safety and reliability of trial results.
2. Define QTLs—identify a limited set of QTLs which focus on factors that are the most critical to quality. The following items should be considered:
  Parameter and description
  Thresholds for the parameter
  Justification for the parameter and threshold
  Action plan in case of deviation from predefined threshold
  Algorithm to calculate the threshold
  Source of data from which to calculate periodic results
3. Develop QTL Monitoring Approach—develop a QTL monitoring plan that includes the following items:
  Timeframe to start and end QTL monitoring
  Frequency of QTL review
  Definition of data sources for QTL monitoring
  Criteria for determining deviation importance
  Action to be taken if important deviations are observed
  Programming and report setup

It is regarded as a best practice to document the details of the QTLs and the QTL monitoring approach in the QTL monitoring plan. This QTL monitoring plan is separate from the study monitoring plan and other trial-level monitoring activities. Note that while it is generally best practice to have QTLs defined prior to the FPFV, time should be allotted for programming, where required.

### Monitor Process Stage

The Monitor stage of the QTL process starts after the first participant has been enrolled. The following steps are included in the Monitor stage (Fig. 4):

- Setup QTL Report—complete programming, configuration, and data mapping activities needed to start QTL reporting for the study.
- Run Report and Assess Result(s)—assess results for the presence of any deviations.
- Investigate Deviation(s)—the following activities could be included when investigating a QTL deviation from the predefined threshold:
  For important deviations that potentially affect participant safety or reliability of trial results, issue management processes may include root cause analysis and determination of corrective or preventive actions.
  For unimportant deviations, incorporate operational changes (within the protocol) or revise the QTL definition or threshold.

### Report Process Stage

The report stage of the QTL process starts after study closure. The following steps are included in the report stage (Fig. 5):

6. Generate QTL Summary Report—the QTL Summary Report is a list of deviations from the defined QTLs and actions taken to address the deviations.
7. Review QTL Deviations—review all QTL deviations from the trial.
8. Report in CSR—summarize the quality management approach and important QTL deviations and actions taken in response in the CSR.

## General Considerations

When implementing QTLs, there are additional important factors to consider as these may influence the overall process.

### Historical Data for Setting Thresholds

Historical data from literature searches or internal company data can be used to help establish QTLs. Sponsors should identify and remove the systematic error and/or bias from historical data used to determine QTLs. Examples of ways to remove systematic errors and bias include the following:

- Excluding significantly outlying studies from the historical data set
- Using the median instead of the mean to define the expected value and minimize the influence of outliers
- Broadening the set of studies in the historical data set if studies of the same type are not available
- Reviewing past quality issues (events) related to QTL deviations
- Using the most recent studies to better represent current practices if the number of historical studies is large
- Considering CRO historical knowledge and/or data

Bias may also arise from the selection of the historical datasets. To avoid bias, the sponsor might consider the following factors when selecting historical data:

- Therapeutic and disease area
- Phase of development
- Patient population (e.g., stage of disease, age, gender, etc.)
- Whether significant process changes have taken place since the historical data was collected
- Assumptions made in prior studies that are not appropriate for the current trial
- Definitions or exclusions of data in an external trial

Finally, in addition to historical data and clinical knowledge, the study team could also leverage other trial-level information from the protocol or the Statistical Analysis Plan (SAP) to predefine QTLs, especially when historical data is not available for a particular type of trial.

Carefully setting the predefined QTL thresholds improves the probability of detection of systemic issues through QTLs.

### Additional Considerations for Defining QTLs

In addition, QTLs fall under the Quality Management section in ICH E6(R2) as a control. This section of ICH E6 notes that the methods used to assure and control the quality of the trial should be proportionate to the risks inherent in the trial and the importance of the information collected. The following information can be considered to assess the level of risk and resulting applicability of QTLs as a control:

1. Trial-level risk management plan (including controls)
2. Number of participants
3. Number of sites
4. Trial Duration—adequate duration of the trial is a consideration to implementing the QTL process and implementing any remedial actions as a part of the QTL process
5. Recruitment rate
6. Trial Design (e.g., dose escalating cohorts because of the small number of participants in each cohort)
7. Trial population

The ICH E6(R2) guideline applies to clinical trial data intended for submission to regulatory authorities. The applicability of QTLs to early phase studies then depends on the clinical development plan for a molecule and whether or not the data is intended for submission. Decisions on the applicability of QTLs and/or the number of QTLs to implement is the responsibility of the sponsor, commonly informed by the study clinician and statistician. At the end of the trial, the quality management approach and any important QTL deviations will need to be documented in the CSR.

### Use of a QTL Library

QTLs are based on the medical and statistical characteristics of a trial; thus, they are inherently trial-specific. However, a QTL library, or set of previously used QTLs and their defining characteristics, can be established as a starting point for QTL definition. While QTLs selected from a library may need to be adapted to trial-specific characteristics, building from common definitions enables later comparison across studies and may streamline the definition process. A QTL library may be built based on therapeutic area or other common clinical trial characteristics. For example, in many oncology studies, parameters focused on Response Evaluation Criteria in Solid Tumors (RECIST) data quality or completeness may be closely related to the interpretability of trial results and applicable to a broad set of studies.

Table 2 is a sample QTL Library with parameters and thresholds for consideration in developing a QTL program. QTL parameters included in the example library are generic and may be applied to studies independent of therapeutic areas.

**Table 2:** QTL Library Example.

Critical to Quality Factor	Parameter	Definition	Justification for Parameter	Tolerance Limit Insights	Comments or Considerations
Signal detection and safety reporting	Percentage or number of immediately reportable events reported late	Percentage or number of immediately reportable events (e.g., SAEs and other events as defined in the protocol) that are reported to the sponsor by the site more than 1 day after investigator awareness of the event	A high number of late reported, immediately reportable events could impact participant safety due to lack of timely awareness of emerging safety profile	Depends on immediately reportable events process at the sponsor company and could be based on analysis of historical data	The metric for this parameter is limited to time of reporting between investigator awareness and submission to sponsor
Eligibility criteria	Percentage or number of participants randomized who do not meet inclusion/exclusion criteria	Percentage or number of randomized participants with Protocol Deviations in inclusion/exclusion criteria	A high number of study participants not meeting the entrance criteria could have a significant impact on interpretation of the primary endpoint and overall validity of the trial results. It can also put participants at undue risk to study drug exposure if they do not meet certain inclusion/exclusion criteria	TransCelerate member company experience suggests that historical data analysis of internal studies is a source of information to establish this threshold. Bias should be evaluated when using any data available in the public domain	The QTL may be particularly relevant to trials with per-protocol analysis. Selection of the most important inclusion/exclusion criteria may be considered for a more focused QTL
Investigational product (IP) handling and administration	Percentage or number of participants with premature discontinuation of IP	Percentage or number of participants who discontinued treatment before the end of the treatment period as defined by protocol	A high number of participants discontinuing treatment could have a significant impact on interpretation of the primary endpoint because of limited/insufficient exposure (the exposure should approximate as close as possible the exposure intended by the protocol)	Very dependent on indication, trial design, and route of administration. Data is publicly available	We acknowledge the participant can discontinue the treatment at any time, but every effort should be made to apply safety management guidelines as this may allow for participant retention on study medication
Withdrawal criteria and trial participant retention	Percentage or number of participants with withdrawal of informed consent	Percentage or number of participants with withdrawal of informed consent after treatment allocation	A high number of participants withdrawing informed consent may indicate excessive patient burden and could significantly impact collection and interpretation of the primary endpoint because of limited/insufficient exposure	Tends to be more specific to burden of the trial procedures Tolerance tends to be lower than losing participants to treatment [5]	In some settings, it may be relevant to consider combining lost to follow-up and withdrawal of consent parameters into a single parameter
Withdrawal criteria and trial participant retention	Percentage or number of lost to follow-up participants	Percentage or number of participants who are lost to follow-up (i.e., have not continued in the trial until the last planned safety assessment and have not revoked consent nor have been reached by investigator site personnel through normal communication methods). These participants have not completed the trial and their status is unknown	A high number of participants lost to follow-up may indicate excessive participant burden and could impact the collection and interpretation of long-term safety and efficacy dat	Tends to be more specific to therapeutic area and long duration studies Tolerance tends to be lower than losing participants to treatment [6]	In some settings, it may be relevant to consider combining lost to follow-up and withdrawal of consent [6]
Procedures supporting study endpoints and data integrity	Percentage or number of study participants for whom study endpoint data was not collected	Percentage or number of study participants for whom study endpoint data was not collected; this could include inability to perform study procedure at the protocol-defined time point	A high number of study participants for whom the failure to collect study endpoint data could impact analysis and interpretation of study results	Tends to be specific to therapeutic area and similarly designed trials	The threshold tends to depend on the trial duration and the burden of procedures to participants
Procedures supporting trial endpoints and data integrity	Percentage or number of participants with important protocol deviations other than eligibility	Percentage or number of participants with protocol deviations during the trial which are not related to protocol inclusion/exclusion criteria (i.e., informed consent, trial intervention, prohibited concomitant medication, trial procedures, safety reporting, and discontinuation)	A high number of study participants with important protocol deviations during the trial could have a significant impact on interpretation of the primary endpoint and overall validity of the trial results. It can also impact participant safety	Therapeutic area specific categorization of important protocol deviations will be trial design specific	Important protocol deviations are defined by the sponsor This QTL may be particularly relevant to trials with per-protocol analysis In the monitoring phase, it should be determined if there is a trend for systematic issues, as multiple types of protocol deviations are captured by this QTL. Some protocol deviations, even if considered important, may not have an impact on the quality of the data It is best to use this QTL in conjunction with other QTLs
Signal detection and safety reporting/procedures supporting trial endpoints and data integrity	Percentage or number of participants on rescue medication	Percentage or number of participants who used a concomitant medication specified in a protocol as a rescue medication	High number of participants who used rescue medication may indicate potential safety issues resulting from an inadequate treatment of an underlying disease. Higher than assumed use of a rescue medication may confound study outcome and introduce bias if it is observed with a higher frequency or duration in one of the study arms. This could have a significant impact on interpretation of the primary endpoint and overall validity of the trial results	Tends to be specific to a therapeutic area and similarly designed trials	The intent of this QTL is to ensure that a protocol is followed and not to limit participants’ access to rescue medication
Investigational product (IP) handling and administration	Percentage or number of participants who are non-compliant with study drug administration as defined in the protocol	Percentage or number of participants whose compliance with study drug is lower than a predefined value	Compliance with study drug which is lower than a predefined value may limit the individual exposure to treatment. High number of participants with low compliance may impact the interpretation of the efficacy results	Tends to be specific to therapeutic area and drug class	Compliance of 80%, for example, means that 80% of individual doses were administered in line with timing and dose requirements specified in the protocol
Procedures supporting trial endpoints and data integrity	Percentage of participants censored in the statistical analysis for primary objective	Percentage or number of participants who are at risk of censoring for primary objective analysis	High number of participants censored for primary objective analysis would translate into smaller than assumed sample size and may impact the interpretation of the efficacy results. Alternatively, final primary objective analysis would be delayed if required sample maturity is not reached within the assumed timelines	Tends to be specific to similarly designed trials and endpoint measures	This parameter is applicable for “time to event” endpoints (e.g., progression-free survival, time to disease relapse/progression). QTLs should be developed with the primary endpoint in mind; QTLs for selected secondary endpoints are up to the discretion of the study team
Randomization	Percentage or number of randomized participants who were incorrectly stratified	Percentage or number of randomized participants who were incorrectly stratified	High number of participants who were incorrectly stratified may lead to imbalances in baseline characteristics between treatment arms, introduce biases in the data, and significantly affect the outcome of a trial	Tends to be specific to similar therapeutic areas and stratification factors	Monitoring of trial performance against the parameter should be based on identification of incorrectly stratified study participants across all study arms (to maintain the blind in case of blinded trial)

The list of parameters included in Table 2 is not intended to be exhaustive. Depending on the trial design, therapeutic area, and indication, parameters from the list may be identified by the study team as applicable to address areas of the highest risk to reliability of trial results and patient safety. Trial-specific QTLs may be added as deemed appropriate by the cross-functional study team.

## Conclusion

Clinical development continues to emphasize risk-based approaches to clinical trial quality. ICH E6(R2) establishes the use of QTLs as a method of risk control to identify systematic issues potentially impacting participant safety or reliability of trial results.

This QTL Framework has been developed to aid clinical development professionals in the implementation of QTLs as part of a broader Quality Risk Management System. The approaches described to manage risk across trial design, conduct, and reporting should benefit sponsors, their vendors, and particularly trial participants in ensuring clinical trials adhere to the principles of GCP and more effectively bring new therapies to those who need them.

## Electronic supplementary material

Below is the link to the electronic supplementary material.

Supplementary file1 (DOCX 15 kb)
