# Supplementary material for: Quality Tolerance Limits: Framework for Successful Implementation in Clinical Development
Source: Ther Innov Regul Sci. 2020 Sep 3;55(2):251–61. doi: 10.1007/s43441-020-00209-0 (PMC7864825; doi:10.1007/s43441-020-00209-0)
Supplement: Supplementary file 1 — Supplementary file1 (DOCX 15 kb) [file 43441_2020_209_MOESM1_ESM.docx]

Callout Box for QTL Manuscript 1

**Key ICH E6 (R2) excerpts related to QTLs**

Section 5.0.4 – Risk Control: “The sponsor should decide which risks to reduce and/or which risks to accept. The approach used to reduce risk to an acceptable level should be proportionate to the significance of the risk. Risk reduction activities may be incorporated in protocol design and implementation, monitoring plans, agreements between parties defining roles and responsibilities, systematic safeguards to ensure adherence to standard operating procedures, and training in processes and procedures.

Predefined quality tolerance limits should be established, taking into consideration the medical and statistical characteristics of the variables as well as the statistical design of the trial, to identify systematic issues that can impact subject safety or reliability of trial results. Detection of deviations from the predefined quality tolerance limits should trigger an evaluation to determine if action is needed.”^1^

Section 5.0.7 – Risk Reporting: “The sponsor should describe the quality management approach implemented in the trial and summarize important deviations from the predefined quality tolerance limits and remedial actions taken in the clinical study report (ICH E3, Section 9.6 Data Quality Assurance).”^1^
